# Mechanisms Involved in the Neuroprotection of Electroacupuncture Therapy for Ischemic Stroke

**DOI:** 10.3389/fnins.2018.00929

**Published:** 2018-12-11

**Authors:** Ying Xing, Min Zhang, Wen-Bin Li, Fang Dong, Feng Zhang

**Affiliations:** ^1^Department of Rehabilitation Medicine, The Third Hospital of Hebei Medical University, Shijiazhuang, China; ^2^Department of Pathophysiology, Hebei Medical University, Shijiazhuang, China; ^3^Department of Clinical Laboratory Medicine, The Third Hospital of Hebei Medical University, Shijiazhuang, China; ^4^Hebei Provincial Orthopedic Biomechanics Key Laboratory, The Third Hospital of Hebei Medical University, Shijiazhuang, China

**Keywords:** electroacupuncture, ischemic stroke, apoptosis, neuroprotection, neurotrophic factors (NTFs)

## Abstract

Stroke is one of the main causes of death all over the world. As the combination of acupuncture and electric stimulation, electroacupuncutre is a safe and effective therapy, which is commonly applied in ischemic stroke therapy in both experimental studies and clinical settings. The review was performed via searching for related articles in the databases of OVID, PUBMED, and ISI Web of Science from their respective inceptions to May 2018. In this review, we summarized the mechanism of EA for ischemic stroke via a series of factors, consisting of apoptosis related-factors, inflammatory factors, autophagy-related factors, growth factors, transcriptional factors, cannabinoid CB1 receptors, and other factors. In summary, EA stimulation may effectively alleviate ischemic brain injury via a series of signal pathways and various other factors.

## Introduction

Ischemic stroke is a primary cause for dependency, disability, and death worldwide (Liao et al., 2017). Occlusion of the middle cerebral artery is the most common cause of ischemic stroke, resulting in high death rates ranging from 40 to 80% (Wicha et al., 2017). Ischemic stroke activates several detrimental cascades that regulate many pathological changes, consisting of apoptosis, excitotoxicity, inflammatory response, and oxidative stress (Li et al., 2017b). Electroacupuncture (EA) stems from the combination of modern electrical stimulation and traditional acupuncture and is a special type of acupuncture (Zhan et al., 2018). Compared to other conventional therapies, EA is widely accepted because it is a relatively safe, cheap, and straightforward therapy (Zhan et al., 2018). EA treatment following ischemic brain injury may produce neuroregenerative or neuroprotective effects via suppressing apoptosis, alleviating glutamate excitotoxicity, enhancing cerebral blood flow and growth factor production, regulating oxidative injury, maintaining blood-brain barrier integrity, and generating cerebral ischemic tolerance (Chang et al., 2018).

## Literature Research Criteria

In this Narrative Review, the MEDLINE (PubMed), EMBASE, Cochrane Central Register of Controlled Trials (CENTRAL) databases (from inception to May 2018) were queried to identify related abstracts and articles. The search terms were “electroacupuncture” and “stroke” or “cerebral ischemia,” and the concerned disease is ischemic stroke.

### The Effect of EA Therapy on the Pathogenesis and Pathological Process of Ischemic Stroke

#### Neurogenesis

Regulation of various growth factors may prompt both neurogenesis and angiogenesis, and improve motor function in the early phases post-stroke (Talwar and Srivastava, 2014). Shin et al. suggest that acupuncture may improve adult neurogenesis via upregulating the expression levels of neurotrophic factors (NTFs) in the brain (Shin et al., 2017). Cheng et al. indicate that the combination of EA and intranasal administration of NGF may effectively promote functional recovery and mitigate ischemic brain injury following focal ischemic stroke via elevating cell proliferation and survival (Cheng et al., 2009). Kim et al. suggest that 2 Hz EA stimulation at Baihui and Dazhui following ischemic brain injury may upregulate endogenous neurogenesis via enhancing differentiation and proliferation of NSCs via the VEGF and brain-derived neurotrophic factor (BDNF) pathway, which contributes to prompting post-ischemic functional recovery (Kim et al., 2014). Tao et al. show that EA intervention with 1–20 Hz following ischemic stroke could increase the proliferation of reactive astrocytes by promoting the expression of crucial trophic factors, such as BDNF, to provide a protective effect against ischemic damage in peri-ischemic regions (Tao et al., 2016).

Kim et al. show that EA at a frequency of 2 Hz and an intensity of 1 mA may induce prominent improvement of motor and neurological functions and exert neuroprotection against ischemic brain injury via elevating the expression of SDF-1α and BDNF (Kim et al., 2013b). EA combined with mesenchymal stem cell transplantation may improve the recovery of neurological function and upregulate the expression of neurotrophic factors, for instance neurotrophin-4/5 (NT4) and BDNF, involved in neurogenesis in mice with ischemic stroke (Kim et al., 2012, 2018). The promotion of NT4 and BDNF secretion in the ischemic brain was accomplished via their common receptor, tropomyosin receptor kinase B (TrkB) (Ahn et al., 2018). In addition, EA may improve synaptic plasticity in rats with ischemic stroke via protecting synaptic ultrastructure and promoting the levels of NGF, BDNF, GAP-43 and p38 in the ischemic brain's cortex (Yi et al., 2006).

Ephrins and Eph receptors are regarded as growth-associated inhibiting factor and are the largest tyrosine kinase family, participating in development processes of the central nervous system. Ren et al. show that EA intervention may improve neural plasticity in peri-ishcemic brain cortexes of rats with acute ischemic stroke, which might involve the enhancement of the ephrin-A5 expression (Liu et al., 2008). Retinoic acid is a crucial regulator of neurogenesis in the hippocampus and subventricular zone. EA may promote neurological deficit recovery via regulation of retinoic acid expression to effectively alleviate ischemic brain damage (Hong et al., 2013). Nestin is a cytoplasmic intermediate filament protein and normally expressed during CNS development (Michalczyk and Ziman, 2005). Ki67, a nuclear protein, is considered as a mitotic marker and expressed at the outset stage of mitosis in the neurogenesis process. EA at 2 Hz may decrease nestin immunoreactive cells and Ki67 in rats after cerebral ischemia/reperfusion (Liao et al., 2017). Heparan sulfate proteoglycans (HSPGs) exist in the extracellular matrix and the cell surface, which is closely associated with synapse function and development (Long et al., 2016). EA treatment can reduce modified neurological severity scores (mNSS) and prompt neural functional recovery following cerebral ischemic stroke via elevating the expression of HSPGs and Slit2 (Long et al., 2016).

GAP-43, as a key factor of axonal growth cones, participates in the process of axonal regeneration (Huang et al., 2017b). EA at acupoints of Neiguan and Zusanli may prompt neural remodeling and improve neural function in cerebral ischemic rats, which is related to promoted expression of GAP-43 surrounding the brain infarction region (Zhou et al., 2011). Nogo protein-A (Nogo-A) is a key myelin-related axonal growth inhibitory protein, mainly inhibiting axonal regeneration after ischemic stroke (Huang et al., 2017b). EA treatment at acupoints along both the Lung Meridian and the Pericardium Meridian may decrease the level of serum Nogo-A in post-stroke rats. Moreover, stimulation in the Pericardium Meridian exerts a better effect than in the Lung Meridian (Chen et al., 2015b). Huang et al. suggest that a treatment of EA at Quchi and Zusanli with 20 Hz for 30 min may promote axonal regeneration via increasing the expression of Gap-43 and decreasing the expression of the Nogo-A signal pathway (Huang et al., 2017b). In addition, the upregulation of phospho-LIMK1 and total LIMK1 levels was related to the synaptic-dendritic plasticity in the hippocampal CA1 region, and EA may down-regulate the expression of miR-134, negatively modulating LIMK1 to elevate synaptic-dendritic plasticity (Liu et al., 2017). Guo et al. show that EA stimulation may significantly increase the expression of IGF-1 mRNA in the striatum and hippocampus, involving the neuroprotective mechanism of EA (Guo et al., 2004). Wang et al. suggest that glia maturation factor (GMF) activation is involved in glial activation. EA intervention is reported to suppress ischemia-elicited astrocyte activation and reduce the expression of GMF. Thus, it is speculated that EA might inhibit astroglial activation via decreasing GMF (Wang et al., 2014) (as shown in Table 1).

**Table 1:** Summary for mechanisms of EA for regulating neurotropic and transcriptional factors against ischemic brain injury.

**References**	**Species/model**	**Acupuncture type/frequency/intensity/time**	**Acupoints**	**Results**
Wang et al., 2003	Wistar rat/MCAO	EA/20–3 HZ/3 mA/30 min	Baihui (DU20) Renzhong (DU26)	Enhancement of the VEGF expression
Pan et al., 2012	SD rat/MCAO	EA/20 HZ/2–4 V/30 min	Hegu (LI4) Quchi (LI 11)	Up-regulation of serum VEGF
Tao et al., 2016	SD rat/MCAO	EA/1–20 HZ/30 min	Quchi (LI11) Zusanli(ST36)	Enhancement of BDNF
Kim et al., 2013b	C57BL/6J/photothrombotic cortical ischemia model	EA/20 HZ/1 mA/20 min	Baihui (GV20) Dazhui(GV14)	Increase of BDNF and SDF-1α
Kim et al., 2012	SD rat/MCAO	EA/3 HZ/until the limb and the ear twitched/5 min	Baihui (GV20)	Motor recovery improvement and BDNF/trkB promotion
Ma and Luo, 2008	Wistar rat/MCAO	EA/40–60HZ/5 V/15 min	Hegu (LI 4)	Angiogenesis factors promotion and anti-angiogenesis factors down-regulation
Zhou et al., 2011	rat/MCAO	EA/20 min	Zusanli (ST36) Neiguan (PC 6)	Promotion of GAP-43
Huang et al., 2017b	SD rat/MCAO	EA/ 20 Hz/30 min	Zusanli (ST36) Quchi (LI11)	Up-regulation of Gap-43; down-regulation of Nogo-A.

Astrocytes are important for the regeneration and recovery of neuronal function and glial fibrillary acidic protein (GFAP) is regarded as a biomarker of astrocytes in the central neuronal system (Seri et al., 2001). High-frequency EA treatment with 15 HZ for 30 min daily for 5 days may decrease the GFAP expression in the hippocampus and the neocortex in rats with post-stroke pain (Tian et al., 2016). EA stimulation with 2 Hz at the acupoints of ST36 and ST37 may improve neurological dysfunction, increase rotarod test times, and decrease cerebral infarct areas. In addition, 2 Hz EA may elevate the number of GFAP immunoreactive cells and decrease nestin immunoreactive cells and Ki67 in rats after cerebral ischemia/reperfusion(Liao et al., 2017). Wang et al. suggest that glia maturation factor (GMF) activation is involved in glial activation, while EA intervention of 5–20Hz for 30 min at Baihui and Shuigou is reported to suppress ischemia-elicited astrocyte activation and reduce the expression of GMF. Thus, it is speculated that EA might inhibit astroglial activation via decreasing GMF (Wang et al., 2014).

#### Angiogenesis

Vascular endothelial growth factor (VEGF) is usually related to angiogenesis formation after cerebral ischemic damage, consequently exerting neuroprotective effects to alleviate the infarct volume (Kim et al., 2018). EA at the acupoints of Baihui and Renzhong induced an elevation of VEGF, which might be associated with a reduction of the infarct area via activating angiogenesis and promoting tissue repair by the proliferation of activating astrocytes following ischemic stroke (Wang et al., 2003). High levels of VEGF induced by EA intervention at Quchi and Zusanli may promote chemotaxis, mobilization and homing of EPCs so as to promote neovascularization (Zhao et al., 2010). EA stimulation with 20 Hz for 30min at both the Pericardium Meridian and the Large Intestine Meridian may elevate VEGF expression and the VEGF-positive microvessel number in ischemic stroke rats, indicating an elevation of cerebral angiogenesis, and stimulation at Quze-Neiguan exerts a better effect than Hegu-Quchi (Pan et al., 2012). Xie et al. show that EA may upregulate and accelerate the formation of stromal cell-derived factor-1α (SDF-1α) concentration gradient and result in the enhancement of angiogenesis and endothelial progenitor cells in an ischemic brain as well as promote the recovery of neurological function (Xie et al., 2016). EA stimulation may promote the expression level of angiogenic growth factors Ang-1 and VEGF and decrease the expression level of endostatin, involved in the process of angiogenesis in ischemic brain tissues from rats (Ma and Luo, 2008) and the mechanism of decreasing ischemic damage (Ma and Luo, 2007).

#### Autophagy

Cytoplasmic light chain 3(LC3) plays a key role in evaluating levels of autophagy (Glick et al., 2010). LC 3-II may be transformed from LC3-I and localizes in autophagosomal membranes, and the number of autophagosomes is closely related to the ratio of LC3-II/ LC3-I (Li et al., 2017b). Liu et al. demonstrate that EA treatment with 1–20 Hz for 3 days may downregulate the ratio of LC3BII/LC3BI as well as decrease the number of autolysosomes, lysosomes, and autophagosomes in the peri-ischemic cortex (Liu et al., 2016a). Furthermore, Li et al. illustrated the activation of autophagy in neurons after ischemia by way of high expression levels of Beclin 1 and LC3 in the peri-infarct cortex(Li et al., 2017b). The expression of autophagosome membrane makers, such as Unc-51-like kinase 1 (ULK1), autophagy-related gene 13 (Atg13) also may be reduced by EA treatment with 1–20 Hz for 3 days after ischemic stroke (Liu et al., 2016a).

The mammalian target of rapamycin (mTOR) kinase produces two functionally different complexes via interacting with other partners, individually named as mTOR complex 1 (mTORC1) and mTORC2; mTORC1 is a main regulator of autophagy and autophagosome formation (Liu et al., 2016a). Formation of autophagosome is regulated by the mTORC1 pathway and LC3-II (Liu et al., 2016a). Liu et al. indicate that EA treatment at the acupoints of ST36 and LI11 significantly upregulated the expression of mTORC1 and the expression of the ULK complex, indicating that the neuroprotection of EA treatment against ischemic injury might be involved in the suppression of autophagy and autophagosome formation (Liu et al., 2016a).

#### Apoptosis

EA of 2 Hz combined with melatonin may exert a neuroprotective effect against apoptosis by improving neurological deficits and infarcted volume, elevating B-cell lymphoma 2 (Bcl-2) and decreasing Bcl2-associated X protein(Bax) (Liu and Cheung, 2013). EA with 5–20 Hz for 30 min once daily at Baihui (DU20) and Shenting (DU24) may enhance Bcl-2 at both protein and mRNA level, phosphorylated-CREB at the protein level, and the activity of glutathione peroxidase and superoxide dismutase in the hippocampus of rats after ischemic stroke (Lin et al., 2015a). On the contrary, EA suppressed the production of Bax and reduced the malondialdehyde level (Lin et al., 2015a). Guo et al. indicate that the neuroprotection induced by EA with taurine might be related to the alleviation of P53 over-expression and up-regulation of the radio of Bcl-2/Bax in ischemic cortexes of rats (Guo et al., 2002). Xue et al. show that EA treatment may contribute to upregulating the expression level of Bcl-xL and anti-apoptotic Bcl-2 in ischemic stroke rats (Xue et al., 2014). Chen et al. demonstrate that EA with 1–20 Hz for 30 min at Quchi and Zusanli may improve cerebral infarction and neural function, and elevate the ratio of anti-apoptotic Bcl-2/Bax in ischemic brain tissue via PI3K/Akt signaling pathway activation (Chen et al., 2012). The neuroprotection of EA preconditioning at the Quchi acupoint against neuronal apoptosis might be partly regulated by the expression of Bcl-2 and Bax via εPKC activation (Wang et al., 2011). EA may increase bcl-2 expression and reduce caspase-3 expression, which might contribute to alleviation of neuronal apoptosis in cerebral ischemia-reperfusion rats (Chen et al., 2009). Xue et al. demonstrated that EA of Zusanli (ST36) and Quchi (LI11) may notably suppress the activities of pro-apoptosis factors, including caspase-3,−8, and−9 (Xue et al., 2014). Wang et al. show that EA may inhibit the activation of caspase-9 and decrease the number of apoptotic cells (Wang et al., 2002).

EA treatment with 4–20 Hz may suppress the death receptor (DR)-mediated apoptotic pathway, which may induce neuronal apoptosis after hypoxia-ischemia, alleviating DR5-induced neuronal apoptosis following ischemic stroke (Xue et al., 2014). Among the members of the apoptosis protein family inhibitor (IAP), anti-apoptotic cIAP-1 and−2 levels were significantly elevated in the cerebral cortex of post-stroke rats after EA treatment (Xue et al., 2014). Shi demonstrates that acupuncture exerts anti-apoptotic effects via inducing the nerve growth factor receptor (trk-A) expression following cerebral ischemic stroke (Shi, 1999) (as shown in Table 2).

**Table 2:** Summary for mechanisms of EA for regulating apoptotic related factors against ischemic brain injury.

**References**	**Species/model**	**Acupuncture type/frequency/intensity/time**	**Acupoints**	**results**
Xue et al., 2014	SD rat/MCAO	EA/4–20 HZ/30 min	Quchi (LI11) Zusanli(ST36)	Elevating the ratio of Bcl-2/Bax
Chen et al., 2012	SD rat/MCAO	EA/1–20 HZ/30 min	Quchi (LI11) Zusanli (ST36)	Enhancement of Bcl-2/Bax ratio
Wang et al., 2011	SD rat/MCAO	EA	Quchi (LI11) Zusanli (ST36)	Up-regulation of Bcl-2; down-regulation of Bax
Liu and Cheung, 2013	SD rat/MCAO	EA/2 HZ/0.5 mA	Zusanli (ST36) xiajuxu(ST39)	Promotion of BCL-2 and Bax
Liu et al., 2016a	SD rat/MCAO	EA/6 V/0.2 mA/30 min	Quchi (LI11) Zusanli (ST36)	Inhibition of LC3B)II/I, ULK1, Atg13, Beclin1; promotion of mTORC1
Wang et al., 2002	MCAO	EA		Activation of the Akt and inhibition of the caspase-9
Xue et al., 2014	SD rat/MCAO	EA/4–20 HZ/30 min	Zusanli(ST36) Quchi(LI11)	Promotion of PI3K, p-Akt, p-Bad and Bcl-2
Chen et al., 2009	SD rat	EA		Up-regulation of bcl-2; down-regulation of caspase-3

#### Inflammatory

1–20 Hz EA treatment were inserted at 2–3 mm depth into Quchi and Zusanli, and may reduce the levels of pro-inflammatory cytokines, including IL-1β, IL-6, and TNF-α, in rats following ischemic stroke (Lan et al., 2013). Acupuncture may alleviate the enhancement of IL-6, IL-1β, and TNF-α, and suppress the microglia activation in ischemic brain tissues, suggesting that the microglia activation may be closely associated with the anti-inflammation effect of acupuncture (Han et al., 2015). EA treatment of 1–20 Hz at Quchi (LI11) and Zusanli (ST36) mediated the miR-9/ NF-κB signaling pathway and decreased the production of the proinflammatory cytokines, IL-1β and TNF-α (Liu et al., 2016c). Huang et al. show that EA treatment with 2–20Hz at Baihui and Shenting may effectively decrease the excessive expression of pro-inflammatory cytokine interleukin-1β (IL-1β) mediated by spinal microglial P2X7R so as to relieve pain hypersensitivity (Huang et al., 2017a). Wang et al. suggest that the mRNA levels of Il6st/Gp130, Cntfr and Lifr were significantly elevated following EA treatment, demonstrating that the IL-6 type cytokines exert a crucial effect in chronic or subacute phases of ischemic stroke following multiple EA interventions (Wang et al., 2014). The combination of EA 2 Hz and melatonin induced an inhibitory effect on COX-2 and TNF-α, playing a key role in neuroprotective effect to alleviate cerebral infarct volume and neurological deficit following ischemic stroke (Liu and Cheung, 2013). Scalp acupuncture may improve ischemic brain injury and inhibit cytokine-mediated inflammation via inhibiting leukocyte infiltration, prompting neurofunctional recovery, downregulating the expression of IL-1β and TNF-α and elevating IL-10 expression in rats (Zhang et al., 2007). Scalp acupuncture with 2–100Hz at the points Baihui and Qubin may elevate TGF-beta1 level and downregulate the expression of NF-κB and COX-2 in cerebrum tissues so as to attenuate ischemic brain injury, inhibiting leukocyte infiltration and improving neurofunctional recovery in rats (Zhang et al., 2009).

MMP-9 and MMP-2, the key members of the MMP family, might result in the degradation of extracellular matrix and aggravate cerebral infarct volume, which are associated with BBB destruction and other pathological processes after ischemic brain damage (Lin et al., 2016). Xu et al. suggest that ischemic brain injury-induced enhancement of MMP2 may be notably alleviated by EA stimulation in ischemic rats (Xu et al., 2014). MMP-2/MMP-9 expression was suppressed by EA treatment in brain ischemic rats and EA may alleviate learning and memory dysfunction as well as anatomical damage in post-stroke rats (Lin et al., 2016). 2 Hz EA at Baihui and Siguan markedly elevated the expression of tissue inhibitors of metalloproteinases-1 (TIMP-1) and reduced the expression of matrix metalloproteinase−9 (MMP-9) at protein and mRNA levels, leading to a balance disorder of MMP-9/TIMP-1expression, which might be involved in neuroprotection caused by EA at the Siguan and Baihui acupoints against ischemic brain injury (Ma et al., 2016).

Cylindromatosis (CYLD) is mainly expressed in peri-infarct cortical neurons, exerting anti-inflammatory and neuroprotective effects in rats after cerebral ischemia/reperfusion (Jiang et al., 2017). Enhancement of CYLD expression induced by EA at the points of Baihui, Hegu, and Taichong may reduce neuronal CX3CL1 expression and inhibit NF-κB nuclear translocation, subsequently suppressing pro-inflammatory cytokines and microglial activation in peri-infarct regions (Jiang et al., 2017). EA exerted anti-inflammatory effects via inhibition of the neuronal NF-kB pathway, which was related to increases of neuronal A20 expression induced by EA treatment in the ischemic brain of rats (Zhan et al., 2016). EA-induced downregulation of the STAT expression following ischemic stroke may exert benefits for rats with chronic and acute ischemic stroke (Su et al., 2012). Wang et al. show that shot single-time EA stimulation at Baihui and Shuigou may significantly upregulate the levels of Stat5a, Stat5b, and Stat6 and multiple EA treatments may remarkably downregulate the levels of Stat1 and Stat2 (Wang et al., 2014). EA may lower the peak expression level of heat shock protein 70 (Hsp70) and adrenocorticotrophic hormone (ACTH) so as to prompt neuronal repair, decrease inflammatory response and suppress excessive stress (Shi et al., 2017) (as shown in Table 3).

**Table 3:** Summary for mechanisms of EA for regulating inflammatory factors against ischemic brain injury.

**References**	**Species/model**	**Acupuncture type/frequency/intensity/time**	**Acupoints**	**Results**
Liu et al., 2016b	SD rat/MCAO	EA/1–20HZ/6 V/0.2 mA /30 min	Quchi (LI11) Zusanli (ST36)	Reduction of IL-1β, IL-6, and TNF-α
Huang et al., 2017a	SD rat/MCAO	EA/2–20 Hz/0.2 mA/30 min	Baihui (DU20) Shenting(DU24)	Down-regulation of IL-1βvia P2X7R
Zhang et al., 2007	SD rat/MCAO	scalp acupuncture		Down-regulation of IL-1β, TNF-α, IL-10
Liu and Cheung, 2013	SD rat/MCAO	EA/2 HZ/0.5 mA	Zusanli (ST36) xiajuxu(ST39)	Inhibition of COX-2 and TNF-α
Wang et al., 2014	SD rat/MCAO	EA/5–20 Hz/1.0–1.2 mA/30 min	“Baihui” (GV 20) and “Shuigou” (GV 26)	Promotion of Il6st/Gp130, Cntfr and Lifr
Zhang et al., 2009	SD rat/MCAO	scalp acupuncture /2–100 HZ/2 mA	Baihui(GV20) Qubin (GB7)	Inhibition of COX-2 NF-kappaB; enhancement of TGF-beta1
Liu et al., 2016c	SD rat/MCAO	EA/4 V/1–20 HZ/30 min	Quchi (LI11) Zusanli (ST36)	Inhibition of IL-1β and TNF-α
Ma et al., 2016	SD rat/MCAO	EA/2 Hz/1 mA/30 min	Baihui(GV20) Siguan	Reduction of MMP-9; Promotion of TIMP-1
Jiang et al., 2017	SD rat/MCAO	EA/1 mA/20 Hz for 5 min, 2 Hz for 30 min	Baihui (GV 20), Hegu(LI4),Taichong (LR 3)	Promotion of CYLD and reducing CX3CL1
Xu et al., 2014	SD rat/MCAO	Acupuncture and EA/2 HZ/1 mA/20 min	Zusanli (ST36) Baihui (GV20)	Reduction of MMP2
Lan et al., 2013	SD rat/MCAO	EA/1–20 HZ/muscle twitch threshold	Quchi (LI11) Zusanli (ST36)	Inhibition of TNF-α, IL-1β, and IL-6.
Lin et al., 2015b, 2016	SD rat/MCAO	EA/20 HZ/1–3 mA/30 min	Baihui (DU20) Shenting(DU24)	Reduction of MMP-2 and MMP-9

#### Blood-Brain Barrier

The blood-brain barrier (BBB) may maintain and protect homeostasis in the CNS and closely govern signaling transmission with the peripheral nervous system (Zhang et al., 2018). EA stimulation provides a beneficial effect in ischemia-reperfusion injured rats via mediating the expression of tight junction proteins, including claudin-5, occludin and ZO-1 (Zhang et al., 2014). EA (100 Hz, 2 mA) of GV 15 and GV 20 with increased NGF levels in the brain may increase BBB permeability in the ischemic cerebral tissue (Lin et al., 2009). EA stimulation at Renzhong (DU26) and Baihui (DU20) may enhance aquaporin-4 expression after ischemic brain damage, which possibly protects the BBB (Peng et al., 2012). Zhang et al. suggested that 8 min duration of EA pretreatment at GV20 and GV26 may significantly enhance exogenous NGF concentrations and increase BBB permeability in the cerebral cortex of rats with ischemic stroke (Zhang et al., 2018). Zou et al. showed that EA preconditioning with a density-sparse wave at Baihui decreases brain edema and BBB permeability by suppressing the expression of p-caveolin-1 and the degradation of tight junction proteins (Zou et al., 2015). EA preconditioning of GV 26 and GV 20 may effectively improve BBB damage via upregulating the level of MMP-9 mRNA, MMP-9 protein and VEGF mRNA after ischemic brain injury (Lin et al., 2015b).

### Different Types of Factors Involved in the Process of EA Therapy Neuroprotection

#### MiRNAs

MiRNAs are a critical class of non-coding RNAs (ncRNAs) composed of 18–24 nucleotides. MiRNAs are important regulating molecules in the etiology and pathology of ischemic brain injury and the percentage of MiRNA changes can be up to 20% in ischemic stroke (Chen et al., 2017). MiR-9 can bind to NF-κB so as to take part in the process of inflammation caused by ischemic brain damage. EA treatment at Quchi (LI11) and Zusanli (ST36) with 1–20 Hz exerts neuroprotective effects via the miR-9-NF-κB downstream pathway after cerebral ischemic injury (Liu et al., 2016c). Zhou et al. show that the neuroprotective effect of EA may be partly reversed by miR-191a-5p, which may aggravate neuronal damage following ischemic stroke. Moreover, reduction of miR-191a-5p in the cortex and primary neurons of rats may improve neurological deficits, decrease infarct volumes, reduce neuronal apoptosis and elevate cell viability (Zhou et al., 2017). Zheng et al. suggest that EA treatment at Neiguan (PC6) and Renzhong (GV26) with a frequency of 2 Hz may reverse the upregulation of rno-miR-494 at 24 h following ischemic stroke. Therefore, rno-miR-494 might be involved in the neuroprotective effect of EA in the process of ischemic brain damage (Zheng et al., 2016). EA may change the expression level of cell proliferation-associated miRNA, including rno-miR-6216, rno-miR-206-3p, rno-miR-494-3p, and rno-miR-3473, which might be related to the improved functional recovery and cerebral blood supply after stroke (Zheng et al., 2016). Hippocampal synaptic plasticity induced by EA was associated with miR-134-mediated LIMK1, involved in the improvement of memory and learning in the period of ischemic-injury recovery (Liu et al., 2017). MiR-181b may be enhanced by EA with 2/10 Hz for 5 successive days in the penumbras, and EA may prompt neurobehavioral function recovery via regulating the expression of RhoA,GAP43, and PirB (Deng et al., 2016).

#### Ion and Ionic Channel Proteins

EA for 30 min/day may protect neurons against ischemic damage via modulating the level of Ca^2+^ to suppress Ca^2+^ overload in the infarct region of the brain (Xu et al., 2002). The large-conductance Ca^(2+)^-activated K^(+)^ (BKCa) expression level was down-regulated by EA at the Shuigou acupoint, which may prompt the recovery of pathological damage and improve neurological deficit in cerebral ischemic rats (Wang et al., 2016). Zhang et al. demonstrate that EA treatment at the Shenting and Baihui for 30 min/day exerts an important therapeutic effect in cognitive recovery, and the key mechanism is related to the regulation of CaM-CaMKIV-CREB (Zhang et al., 2016). EA treatment may effectively suppress the activity and expression level of CaM, subsequently increasing the CREB and CaMKIV expression (Zhang et al., 2016). Ren et al. show that EA treatment may regulate the expression of Na(v)1.6 and Na(v)1.1 following ischemic stroke, which may be related to the neuroprotection mechanism of EA treatment (Ren et al., 2010b). EA stimulation (density wave, frequency 10 Hz and intensity 1 mA) at PC6, SJ5, SP6, and ST36 may modulate the expression of Nav1.1 following acute ischemic stroke, which may be involved in the protective mechanism of EA treatment for ischemic brains (Ren et al., 2010a). Sun et al. show that transient receptor potential melastatin7 (TRPM7) plays an important role in the process of ischemic stroke, and EA may reverse the up-regulation of TRPM7 expression in cerebral infarction rats. The pathway of trkA might be involved in the effect of EA on TRPM7 (Zhao et al., 2005). Moreover, the expression level of the water channel proteins, AQP9 and AQP4, may be markedly inhibited after EA treatment with a 2 Hz frequency (intensity, 1 mA) at GV20 and ST36 in the infarct brain, demonstrating that the neuroprotection mechanisms of EA are partially involved in the improvement of inflammation-mediated cerebral edema (Xu et al., 2014).

#### ACh and Related Receptors

Kim et al. indicate that EA stimulation at a 2 Hz frequency of 1 mA intensity for 20 min may significantly increase the release of acetylcholine (ACh) from the cholinergic nerve in the ischemic brain cortex. ACh upregulation contributes to the release of NO from endothelial cells, and the over-expression of NO leads to vasodilation (Kim et al., 2013a). EA treatment at 2/15 Hz sparse-dense frequency and an intensity of 1 mA for 30 min may significantly reverse downregulation in the mRNA level of α7nAChR, choline acetyltransferase, and five subtypes of muscarinic receptors, subsequently alleviating damage of the central cholinergic system (Chi et al., 2018).

#### Nitric Oxide Synthase (iNOS)

Shi suggests that enhancement of NO levels in peri-infarction is closely relevant to iNOS immunoactivity and acupuncture may suppress iNOS immunoactivity via reducing the NO content in ischemic stroke rats (Shi, 1999). The effect of EA stimulation at Baihui and Dazhui with 2 Hz for 20 min on moderate ischemic damage is completely inhibited in eNOS KO mice, demonstrating that the neuroprotection of EA is closely related to eNOS (Kim et al., 2013a).

#### Nogo Protein and Its Receptors

In the spinal cord, the medulla oblongata and the cerebral cortex of ischemic stroke rats, upregulation of Nogo-66 receptor (NgR) expression is a critical cause of remote-organ damage of acute cerebral ischemia (Tan et al., 2014b). The neuroprotective effect induced by EA at Baihui (GV 20), Shuigou (GV 26), and Neiguan (PC 6) might be significantly associated with suppressing the expression level of NgR protein, which is the receptor of myelin growth suppression regulator Nogo-A in the brains of rats with hypertensive ischemic brain damage (Tan et al., 2014b). Tan et al. demonstrate that the up-regulation of NgR1 and Nogo-A induced by ischemia may be reversed by EA treatment at days 14 and 28 following ischemic stroke in renovascular hypertensive rats, and EA may alleviate neural damage following cervical spinal cord injury (Tan et al., 2014a). Deng et al. show that PirB protein and pirb mRNA levels in the penumbra are reduced by EA treatment at a dense-disperse frequency of 2/10 Hz and an intensity of 1–2 mA at the Baihui point within 28 days following ischemia/reperfusion, and the decrease of pirB might promote neurite outgrowth following oxygen-glucose deprivation damage (Deng et al., 2016).

#### MAPK Signal Pathway

EA treatment may suppress the nuclear translocation of NF-κBp65 and the expression of MyD88 and p38 MAPK so as to inhibit the production of pro-inflammatory cytokines, which is a possible mechanism for explaining how EA ameliorates the excessive activated microglia (Liu et al., 2016b). Liu et al. suggest that EA treatment at Zusanli and Quchi with dense disperse waves of 1–20 Hz may suppress nuclear translocation of NF-κB p65 in the peri-ischemic cortex of rats following ischemic stroke (Liu et al., 2016c). Systemic EA stimulation with 1–20 Hz at the points of Zusanli and Quchi may suppress the activation of the TLR4/NF-κB pathway after ischemia/reperfusion injury by decreasing crucial target points of the TLR4/NF-κB signaling pathway (Lan et al., 2013). As two crucial downstream target points of the NF-κB signaling pathway, pro-apoptotic Fas, and Bax are significantly suppressed by EA treatment. The protection mechanisms of EA at Shenting and Baihui on the improvement of cognitive impairment might be involved in the suppression of NF-κB-mediated cell apoptosis in ischemic/reperfusion rats (Feng et al., 2013).

Huang et al. show that EA stimulation may provide protection against ischemic cerebral damage via activating the ERK1/2 pathway (Huang et al., 2014). EA notably elevates the expression of ERK phosphorylation so as to prompt cerebral cell proliferation, and the expression levels of cyclin-dependent kinase (CDK)4 and cyclin D1 are also enhanced via ERK activation in ischemic brain tissues (Xie et al., 2013).

EA intervention with disperse waves of 4 and 20 Hz for 3 days may activate the PI3K/Akt signal pathway, which might be associated with the anti-apoptotic form of IAP(inhibitor of apoptosis protein) and the Bcl-2 (Xue et al., 2014). Activation of the PI3K/Akt signaling pathway induced by EA intervention may be involved in EA's mechanism against ischemic brain injury (Sun et al., 2005). Furthermore, as the PI3K activators, EA may upregulate the serum levels of GDNF and BDNF (Chen et al., 2012). Li et al. show that EA stimulation at GV26 may partially reduce the enhancement of AngII and its receptor-regulated IP3 signal pathway induced by ischemic stroke, and then improve blood supply and decrease vasoconstriction in the infarct region, providing a neuroprotective effect in ischemic rats (Li et al., 2014).

Zhao et al. demonstrate that EA stimulation with disperse waves of 4 and 20 Hz for 21 days may prompt the neurogenesis of NSCs via enhancing the Notch1 expression level after ischemic brain injury (Zhao et al., 2015). EA therapy with frequencies of 1 or 20 Hz for 30 min/day markedly suppresses the transcription of GSK3 and upregulates the expression of β-catenin and Wnt1 in neural progenitor cells (NPCs), suggesting that EA may prompt the NPCs' proliferation in the peri-ischemic cortex by the Wnt/β-catenin pathway at the Zusanli (ST36) and Quchi (LI11) points (Chen et al., 2015a). Wu et al. show that the phosphorylation level of AMP-activated protein kinase α (AMPKα) in motor cortex, somatosensory cortex and caudate putamen regions may be elevated by EA, promoting motor functional recovery and neural activity following ischemic brain injury (Wu et al., 2017).

#### Glutamate and Its Receptors

Glutamate accumulation occurs immediately following ischemic stroke, leading to an excessive enhancement of glutamate receptors and resulting in neurotoxicity (Liu et al., 2015). Yue et al. suggest that EA and acupuncture treatment may upregulate the serum gamma-aminobutyric acid (GABA) levels and decrease serum glutamate (Glu) levels as well as the ratio of Glu/GABA compared to those before treatment in patients with stroke, but EA exerts a better effect on those than the acupuncture group (Yue et al., 2012). Zhang et al. demonstrate that acupuncture (twice daily for 1 week, 20 min for each time) at Baihui may prevent damage of synaptic transmission and spike encoding at GABAergic neurons from ischemic stroke, and the preventive effect is related to the resistance ability of GABAergic cells to changes in refractory periods and changes of spike threshold potentials following stroke (Zhang et al., 2011). Shi shows that acupuncture may decrease the N-methyl-D-aspartate receptor subtype 1 (NMDAR1) mRNA mediated excitotoxicity of glutamate after cerebral ischemia-reperfusion injury (Shi, 1999). EA stimulation with dense-sparse frequencies (sparse 4 Hz for 1.5 s and dense 16 Hz for 1.5 s alternately) may increase the expression of TrkA and reduce the over-expression of the NMDAR1 subunit in MCAO rats (Sun et al., 2005). EA with 2 Hz may improve long-term potentiation and behavior impairment after ischemic stroke via reversing the upregulation of transient receptor potential vanilloid subtype 1 (TRPV1) and NMDAR1 in the hippocampal CA1 regions (Lin and Hsieh, 2010). EA stimulation may reverse the promoted level of NMDAR1 mRNA induced by ischemic stroke (Sun et al., 2005). Zhao et al. show that a bidirectional mediation of extracellular inhibitory and excitatory amino acid (glutamate, aspartate, and taurine) levels may be involved in EA, with 15 HZ induced neuroprotection following ischemic stroke (Zhao and Cheng, 1997).

#### Protein Kinase C

Expression of protein kinase C (PKC) in the vascular smooth muscle of the focal ischemic brain was notably suppressed by EA treatment following cerebral infarction, which might be involved in the protective mechanism of EA for ischemic stroke (Xu et al., 2012). EA treatment (15 Hz, 1 mA) at Shuigou may improve acute cerebral infarction via relieving arterial spasm in acute cerebral infarction rats via up-regulating the activity and immunoactivity of PKC in the vascular smooth muscle of the middle cerebral artery (Lü et al., 2015).

#### 5-Hydroxytryptamin

The possible mechanism of post-stroke insomnia recovery caused by low-frequency EA intervention may be involved in the down-regulation of plasma norepinephrine and the upregulation of plasma 5-hydroxytryptamine (5-HT) (Tang et al., 2015). Head point-through-point EA therapy may effectively treat post-stroke depression via significantly upregulating plasma 5-HT levels in patients (Xue et al., 2007).

#### Cannabinoid CB1 Receptors

In the central nervous system, endocannabinoids exert a series of different functions through the significantly localized stimulation of cannabinoid CB1 receptors (CB1R) in the cortex, basal ganglia and hippocampus (Liu et al., 2015). Tian et al. suggest that delta-opioid receptors may be up-regulated by EA stimulation at Shuigou and Neiguan for 30 min/day so as to protect the brain against ischemic brain injury (Tian et al., 2008). Enhancement of glutamate receptor subunit 2 (GluR2) induced by EA with 2–15 HZ for 30 min/days exerts a neuroprotective effect against global cerebral ischemia via CB1R, providing a new possible therapy target (Liu et al., 2015).

#### Cell Proliferation Related Molecules

EA at the point of Quchi and Zusanli may enhance the expression of p-Rb, CDK4, and CyclinD1, and BrdU labeling in the peri-ischemic striatum and cortex after stroke (Tao et al., 2016). EA at Zusanli and Quchi with 1–20 Hz may lead to promotion of the gene and protein expression level of cyclin E, CDK2, CDK4, and cyclinD1, shortening the G1-phase, omitting the G0/S and/or G1/S transition point and resulting in continued proliferation (Huang et al., 2014). EA treatment notably decreases the negative regulators p27Kip and p21Cip1 in the protein and gene levels, which may effectively reverse the effect of these factors in suppressing positive regulatory factors, thus enhancing cell proliferation (Huang et al., 2014).

#### Transcriptional Factors

EA treatment could reduce the neurological score, alleviate neurological dysfunction in rats following ischemic stroke, and promote the expression level of Slit 2 and Robo 1, which might be involved in the mechanism of EA treatment for alleviating brain infarction in the clinic (Lu et al., 2013). EA intervention at acupoints alleviates the downregulation of cell division-cycle 42 (Cdc 42) and upregulation of Slit-Robo GTPase-activating protein-1 (srGAP 1) caused by brain ischemia in rats, demonstrating that EA could exert benefits in neurological function following ischemic stroke (Lin et al., 2016). Lu et al. show that EA promotes the expression of Monocarboxylate transporter 1 (MCT1) in astrocytes and enhances the transportation of lactate from astrocyte to neurons as energy substrates (Chen et al., 2015b). EA (15 Hz, 2 mA) plays a key role in establishing collateral circulation and blood vessel regeneration via enhancing the expression level of Apelin-APJ for vascular endothelial cells in ischemic rat brains (Yang et al., 2017). Li et al. show that EA may raise hypoxia-inducible factor-1α (HIF-1α), reduce cerebral IV and promote recovery of neurological function in cerebral ischemic rats (Li et al., 2017a). After damage of cerebral tissue, abnormal activated astrocytes will prompt the synthesis and release of pain signal transduction-associated mediators, for example cyclooxygenase-2 (COX-2). COX-2 expression was markedly upregulated in the damaged hippocampus of animal models after global cerebral ischemia, and EA may exert an analgesic effect via reducing COX-2 expression in the hippocampus (Tian et al., 2016).

#### Serum Proteins

Pan et al. show that EA may change the expression level of multiple serum proteins, including upregulation of gelsolin, beta-2-glycoprotein I proteins, C3, C4B, and complement component I, and downregulation of SerpinG1 protein in acute ischemic stroke patients (Pan et al., 2011). EA at Shenting and Baihui with 1–20Hz for 30 min daily for 7 days may downregulate the expression of Ras homologous member A, and upregulate the expression of F-actin proteins, Ras-related C3 botulinum toxin substrate 1 and cell division cycle 42, Which demonstrates that dendritic spine plasticity and Rho GTPases play a key role in the mediation of EA effects related to cognitive function recovery after ischemic brain injury (Lin et al., 2016). Suppression of platelet-associated complement-1 (PAC-1) induced by EA at yangning meridian acupoints may promote the recovery of paralyzed lower extremities in patients with acute ischemic brain injury (Tang et al., 2015).

## Conclusion

**Figure 1:**
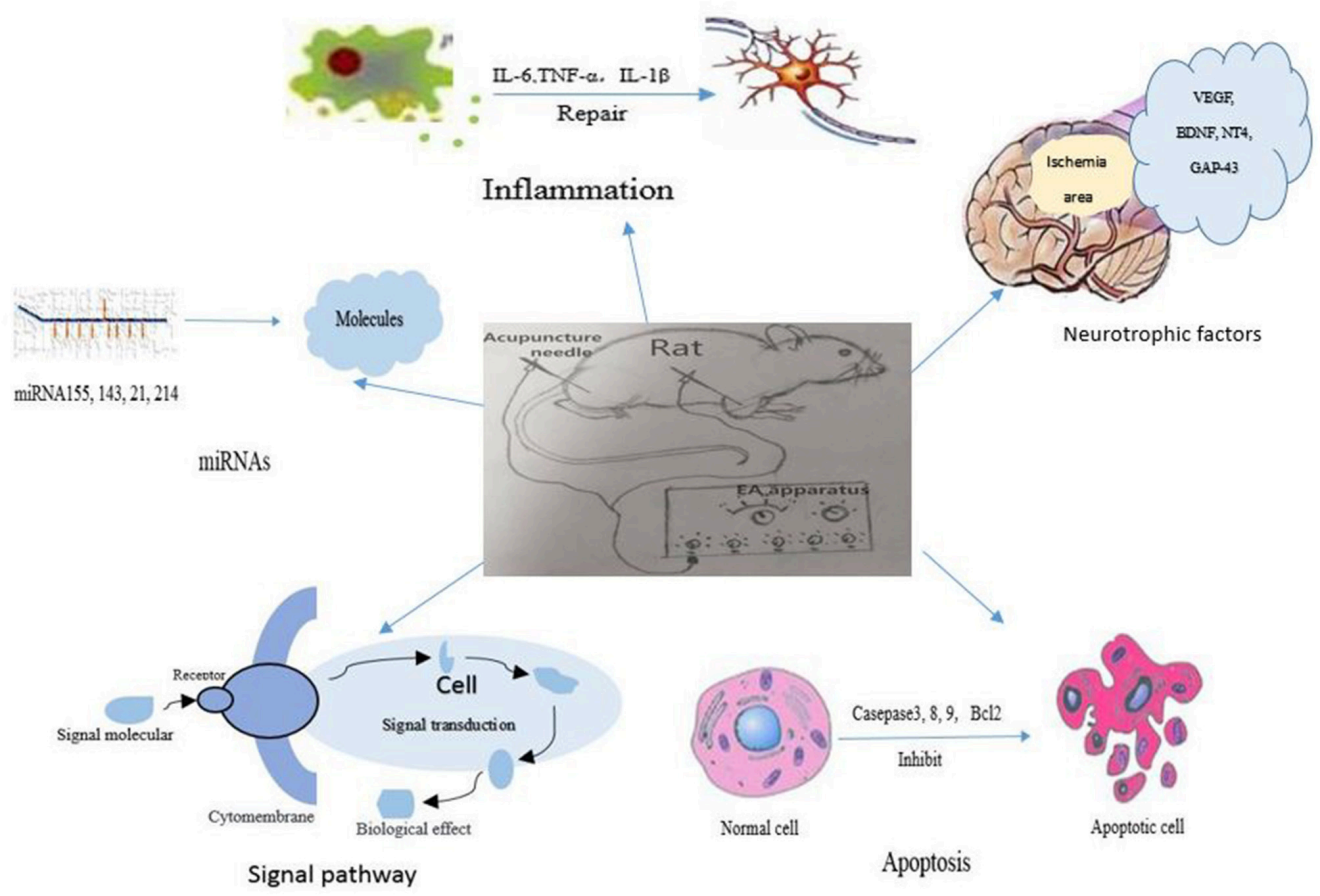
The signal pathways and factors involved in the mechanisms of electroacupuncture therapy for ischemic stroke.

In recent years, EA has not only been applied as a supplementary treatment for post-stroke recovery but also as a preventive intervention for stroke. EA has shown neuroprotective effects in both animal studies and clinical trials. There is growing evidence that EA attenuates ischemic brain injury via regulating considerable molecules, for instance apoptosis-related factors, inflammation-related factors, autophagy-related factors, glutamate and its receptors, miRNAs, neurotropic, and transcriptional factors, becoming involved in different signal pathways, as shown in Figure 1. Meanwhile, the clinical effects of EA included life quality improvement, attenuation of pain, enhancement of cerebral blood flow and daily-life activity promotion in stroke patients. These beneficial effects of EA might be closely related to the above-mentioned neuroprotective mechanisms which were confirmed in animal studies. Moreover, the potential adverse effects of EA should be attached with enough importance. If the needle is not disposable, the contaminated needle might help the spread of infectious diseases; if the practitioner is not skillful enough, the needle might damage the internal organs in the abdomen; if the treatment point is not selected appropriately, the needle might hurt the vascular system or nerves in the respective region of body.

In conclusion, EA stimulation is a safe and effective therapy in reducing ischemic brain injury. The mechanisms for neuroprotection of EA might explain the why EA may exert benefits for stroke patients in clinical settings. Further research on molecular mechanisms of EA might provide therapeutic targets as well as a more optimized strategy for acupoint selection and therapy dosage.

## Author Contributions

YX and FZ conceived the main ideas and wrote the manuscript. W-BL, MZ, and FD searched the references and designed the framework.

### Conflict of Interest Statement

The authors declare that the research was conducted in the absence of any commercial or financial relationships that could be construed as a potential conflict of interest.
